# Perceived Peer Relationships in Adolescence and Loneliness in Emerging Adulthood and Workplace Contexts

**DOI:** 10.3389/fpsyg.2022.794826

**Published:** 2022-06-10

**Authors:** Chi Chiao, Kuan-Chen Lin, Laura Chyu

**Affiliations:** ^1^College of Medicine, Institute of Health and Welfare Policy, Institute of Public Health, National Yang Ming Chiao Tung University, Taipei, Taiwan; ^2^Department of Family Medicine, Taipei Veterans General Hospital, Taipei, Taiwan; ^3^Master of Public Health Program, School of Nursing and Health Professions, University of San Francisco, San Francisco, CA, United States

**Keywords:** loneliness, perceived peer relationships, workplace, life course, childhood circumstances, young adults (18–34 yrs), Taiwan youth project

## Abstract

**Background:**

A common life-course hypothesis is that negative early-life experiences contribute to poor health in later-life. However, little is known about perceived peer relationships during adolescence and the feeling of loneliness in emerging adulthood. This study explores the perception of adolescent peer relationships in a school context and its association with loneliness in adulthood and in workplace contexts.

**Methods:**

This study used data from a cohort sample of 2,520 adolescents from the Taiwan Youth Project (*N* = 2,520), consisting of eleven waves of data collected from 2000 to 2017. Major measures included the Loneliness Scale (6-item de Jong Gierveld short scale) and perceived peer relationships (classroom cohesion and perceived popularity among classmates) in middle school. Multivariate multinomial logistic regressions were used to estimate the associations of perceived peer relationships during adolescence and workplace characteristics with loneliness in adulthood.

**Results:**

Positive perceived peer relationships in adolescence were significantly related to decreased risk of serious social loneliness [Relative risk ratios (RRR) 0.70, 95% CI: 0.58–0.85] and severe social/emotional loneliness (RRR = 0.76, 95% CI: 0.63–0.91) in adulthood. Workplace satisfaction was a protective factor of severe social/emotional loneliness in employed adults.

**Conclusion:**

Adolescents who perceived peer relationships in middle school as positive were less likely to report social and emotional loneliness during adulthood. Satisfaction in the workplace characteristics was also associated with lower risk of loneliness in adulthood. Theoretical and policy implications are discussed.

## Introduction

Despite global support for improving the psychological well-being of adolescents over the past few decades, youth psychological well-being, including loneliness, still remains a great concern (Cacioppo and Cacioppo, 2018). In addition to Rook’s definition of loneliness as *an enduring condition of emotional distress that arises when a person lacks appropriate social partners for desired activities, particularly for activities that provide a sense of social integration and opportunities for emotional intimacy* (Rook, 1984), loneliness is interpreted as a condition wherein mismatch occurs between personal social needs and perceived social interactions (Rook, 1984; McWhirter, 1990; Schwartz-Mette et al., 2020). Prior studies have found loneliness to be perceived isolation that has negative physical and mental outcomes (Cacioppo and Hawkley, 2009). For instance, research suggests that loneliness may be associated with poor psychological well-being, including self-esteem (Cacioppo et al., 2006; Miller, 2011; Musetti and Corsano, 2021), as well as fatigue and mortality (Davies et al., 2021). Lonely people were found to be more likely to focus on negative cues in their social relationships, which in turn negatively impacts health and leads to a vicious cycle (Cacioppo and Cacioppo, 2018).

A large body of evidence supports the long-term impact of early life experience on psychological outcomes (Hyland et al., 2018; Lin and Chiao, 2020; Musetti et al., 2021b). The adolescent years involve formulating a social identity, which has long been recognized as an important developmental stage and largely determined by a range of influences that includes parents, peers, and school (Musetti et al., 2021a). Adolescence is a critical period that shapes how relationship characteristics, particularly peer relationships, are longitudinally linked to the development of psychological well-being during adult life (Miething et al., 2016), including two domains of loneliness. Emotional loneliness is related to the lack of an intimate relationship such as partner and best friend; social loneliness is referred to as the lack of a broader, engaging social network, including families, relatives, friends, and neighbors (Weiss, 1973). Perception of peer relationships during adolescence is hypothesized to be an important source of developing social relationships as well as affecting loneliness in later life.

Research has identified various factors associated with a range of domains of loneliness (Hyland et al., 2018; Chiao et al., 2019; Lin and Chiao, 2020). These factors include individual socio-economic characteristics, such as age, gender, and work characteristics, partnership status, individual self-esteem (Mund et al., 2020), and family cohesion (Fujimori et al., 2017; Chiao et al., 2019). Relatively few studies have highlighted the importance of perceived peer relationships, popularity among classmates, and academic performance (Mouratidis and Sideridis, 2010; Putarek and Keresteš, 2016; Schwartz-Mette et al., 2020). Accordingly, the role of such relationship factors in adolescence and how they affect loneliness are under-explored. Moreover, little is known about peer influences in school contexts during adolescence and their prospective link to loneliness during young adulthood. This is particularly true in the context of Asian countries, including Taiwan.

The school attachment hypothesis (Dornbusch et al., 2001; Denny et al., 2011) provides a strong theoretical framework for this study. School attachment refers to connectedness between the school environment and its members. Interactions with peers and families in the school environment are proposed to produce cumulative social exposure in a context where the adolescent is growing and living (Dornbusch et al., 2001). Peers in the present study are referred to as classmates and their relationships are likely to have both inhibiting and activating effects on psychological well-being. Due to education policies, each middle-school student in Taiwan has his or her homeroom, in which they spend most of their school hours. Classrooms in middle and high schools thus are an important environment in Asian schools for the development and establishment of peer relationships. The perceived peer relationships in such classroom contexts include peer acceptance and popularity. Class cohesion specifically represents acceptance among classmates (Dornbusch et al., 2001; Denny et al., 2011; Schwartz-Mette et al., 2020).

In their 30s, many individuals are getting married, becoming parents, and developing their careers. From a life course perspective, entrance into employment is an important milestone in adulthood that warrants attention. Yet, there is limited research on work and workplace characteristics and their association with loneliness during adulthood. Studies have suggested that occupational loneliness is not only related to the work context, such as wages, work benefits, and occupational sector, but also to the individual’s social environment (Dornbusch et al., 2001; Fernet et al., 2016; Gunes and Bilek, 2020). Social companionship and emotional deprivation at work have been found to be underlying factors for loneliness (Creed and Reynolds, 2001; Wright, 2005). Our study examines if workplace characteristics are associated with loneliness among employed participants.

Prior research has demonstrated that specific indicators of loneliness tend to cluster within certain loneliness domains (Hyland et al., 2018; Chiao et al., 2019; Lin and Chiao, 2020). We have extended this line of inquiry and created a representation of the latent structure of the loneliness during adulthood. Such a latent structure consists of multiple clusters that characterize the underlying relationships of social and emotional loneliness domains. As suggested by prior research, these identified clusters may include non-loneliness, emotional loneliness, social loneliness, and both emotional and social loneliness (Hyland et al., 2018; Chiao et al., 2019). Notwithstanding the above findings, empirical research on loneliness clusters has not adequately explored their associations with perceived peer relationships.

To bridge the knowledge gap, we explore perceived peer relationships during early adolescence and its prospective association with loneliness clusters during emerging adulthood, with a focus on school and workplace contexts. We leverage longitudinal data from a Taiwanese sample to examine two research questions. First, what aspects of perceived peer relationships in adolescence increase the risk of loneliness in young adulthood? We specifically examine classroom cohesion and popularity and their association with loneliness clusters in adulthood. Second, what workplace factors influence employed young adults’ risk of loneliness? We explore how workplace characteristics in adulthood, including workplace satisfaction, work-life balance, and frequency of alcohol consumption on the job, may contribute to loneliness among employed persons. These workplace characteristics are hypothesized to affect social companionship and emotional deprivation at work (Wright, 2005; Wootton et al., 2021). We also explored whether there is a workplace context pathway by which perceived peer relationships in adolescence have an effect on loneliness clusters during adulthood. We proposed the following hypotheses:

**H1**. Positive perception of peer relationships in adolescence, namely classroom cohesion and popularity among classmates, would be negatively associated with adult loneliness in emotional and social domains.

**H2**. Supportive workplace characteristics in adulthood, including workplace satisfaction, work-life balance, and less drinking on the job, would be negatively related to adult loneliness among the employed sample.

**H3**. The association between workplace characteristics and adult loneliness would be conditional on the level of perceived peer relationships in adolescence.

## Materials and Methods

### Participants

The dataset used in this study is from the Taiwan Youth Project (TYP), which recruited a cohort of junior high school students from the 2000 and 2002 classes. These students were 13–15 years old and from Northern Taiwan. The study was initiated in 2000 and had eleven follow-ups in 2001, 2002, 2003, 2005, 2006, 2007, 2009, 2011, 2014, and 2017. The TYP survey used a multi-stage random sampling framework to obtain school-based representative samples of middle school students in Northern Taiwan, including Taipei City, New Taipei City, and Yi-Lan County. Additional details of the sampling design and data collection procedures are described elsewhere (Chiao et al., 2019; Lin and Chiao, 2020).

The TYP surveys provide longitudinal information on a range of school, family and demographic variables from early adolescence to emerging adulthood. To achieve the research objectives, waves of data used were primarily when participants were adolescents (waves 1 and 2 in 2000–2001), and adults in their 30s (wave 12 in 2017). We restricted our analyses to participants with complete responses for the major measures collected in 2017 (wave 12). This yielded an analytical sample of 2,520 young adults and a subsample of 2,287 working adults. We assessed differences in individual characteristics between employed and unemployed samples. Difference in distributions between the total and the employed samples indicated that the employed sample was more likely than the unemployed sample to be male gendered, have a partner, and have a higher level of classroom cohesion and family cohesion.

The TYP dataset is publicly available and can be used for research with the approval of Academia Sinica in Taiwan.^1^ All TYP participants gave informed written consent at the start of their interviews. The study protocol was approved by the Research Ethics Committee of National Yang Ming Chiao Tung University (Taipei, Taiwan) (IRB Number: YM106103E-2).

### Measures

#### Loneliness Clusters

The outcome variable of loneliness during adulthood was measured by the self-reported 6-item de Jong Gierveld Scale (DJGS), which includes emotional and social dimensions (Weiss, 1973; Rook, 1984; McWhirter, 1990; De Jong Gierveld and Van Tiburg, 2010) in wave 12. The DJGS scale consists of a 3-item emotional scale (“There are plenty of people I can rely on when I have problems,” “There are many people that I can count on completely” and “There are enough people that I feel close to”) and a 3-item social scale (“I experience a general sense of emptiness,” “I miss having people around” and “Often, I feel rejected”). These items have three response categories: “no,” “more or less”, and “yes.” Based on prior studies (Dornbusch et al., 2001; Chiao et al., 2019; Lin and Chiao, 2020), each item was recoded into a dichotomous score, indicating whether subjects were extremely lonely (coded as 1) or not lonely (coded as 0). We further conducted latent class analysis (LCA) to estimate the probabilities of individual young adults clustered in a number of loneliness domains (Hyland et al., 2018; Chiao et al., 2019; Lin and Chiao, 2020). Informed by model fit indices (AIC and BIC), along with conceptual interpretations, the LCA results yielded three mutually exclusive groups: 44% in the non-loneliness group (reference group), 25% in the serious social loneliness group, and 31% in the severe emotional/social loneliness group. Recommended by prior studies (Hyland et al., 2018; Chiao et al., 2019), the non-loneliness group consisted of individuals with an average score within one standard deviation below the sample mean. The group of severe emotional/social loneliness consisted of young adults who were more likely to report social and emotional loneliness. In contrast, the group of serious social loneliness included young adults who were more likely to report social loneliness. The label of *severe* captures greater intensity than the label of *serious* (Appendix 1).

#### Perceived Peer Relationships

The primary predictor variable of perceived peer relationships was operationalized by classroom cohesion and adolescent popularity (waves 1 and 2 in 2000–2001). Classroom cohesion was the average of three items: (1) “My classmates are always willing to help whenever I need them”; (2) “Our classmates are close to one another as if we were a family”; and (3) “I like to interact with my classmates.” The respondents rated each item on a 4-point scale, with a higher score representing a more cohesive relationship (Cronbach’s α = 0.65) (Yi et al., 2009). Adolescent popularity was assessed by respondents’ perceptions of their popularity during middle school. This information was obtained from the question, “Are you worried about not being popular?” Responses were on a 4-point scale, and ranged from strongly disagree to strongly agree. Research suggests that academic performance is related to classroom popularity (Alon, 2009), especially in Asian countries (Yi et al., 2009). We included an academic performance variable operationalized as individual’s average academic score in the previous semester. Responses were categorized ordinally to five categories: top 5 in the class; rank 6–10; 11–20; 20–30; and below 30 in ranking.

#### Family Background

Family characteristics consisted of family cohesion during adolescence, (waves 1 and 2 in 2000-2001), and choice of partnership during young adulthood (wave 12 in 2017). Family cohesion measure used a self-reported 4-item scale, with a higher score representing stronger family cohesion (Cronbach’s α = 0.78) (Yi et al., 2009; Chiao et al., 2019). Choice of partnership during adulthood was grouped into three categories (single without a partner, single with a partner, and ever married).

#### Psychological Well-Being

Measures of psychological well-being during adolescence included self-esteem and feelings of being lonely when attending middle school in 2000-2001 (waves 1 and 2). Self-esteem was assessed by six items of the Rosenberg’s Self-Esteem Scale (Cronbach’s α = 0.72) (Rosenberg, 1979). Higher scores represent higher level of self-esteem. Feelings of loneliness were measured by asking adolescents if they had felt lonely during the past two weeks. Responses were dichotomized as slightly serious, serious, and very serious feelings of being lonely (coded as 1)”; and, “no feelings of being lonely (coded as 0)” (De Jong Gierveld and Van Tiburg, 2010; Lin and Chiao, 2020).

#### Work Characteristics

Workplace characteristics during adulthood were assessed by multiple measures in 2017 (wave 12). Respondents first reported whether they were currently employed. The subjects were then asked about levels of satisfaction about work (work environment, job duties, and working hours), the benefits of their employment (compensation/salary, employee benefits, and promotion opportunities), and work-related social companionship (with co-workers and supervisors). Responses ranged from very dissatisfied (coded as 1) to very satisfied (coded as 4). In addition, the subjects were also asked whether or not they needed to work at night or on holidays (yes/no), and frequency of alcohol drinking for work purposes, ranging from never (coded as 0) to daily (coded as 4). Prior studies have shown that alcohol consumption is significantly related to loneliness (Stickley et al., 2014; Chiao et al., 2019). Yet, little is known about whether alcohol drinking related to work is associated with loneliness. In addition to general workplace characteristics, we also specifically addressed behavioral aspect of work characteristics.

### Statistical Analyses

To examine whether perceived peer relationships with classmates in adolescence are longitudinally associated with loneliness during adulthood, we employed a two-part model analysis. The first part assessed whether perceived peer relationships in adolescence were associated with adult loneliness among all 2,520 young adults. We adopted multinomial regression techniques to estimate the likelihood of being within a certain loneliness cluster with respect to perceived peer relationships, academic performance, family cohesion, and other related characteristics.

In the second part of the analysis, we created a subsample of 2,287 employed individuals from the total sample to determine whether workplace characteristics during adulthood were associated with loneliness, adjusting for perceived peer relationships during adolescence. To explore whether the effect of workplace characteristics on loneliness varied by perceived peer relationship during adolescence, we assessed interactions between workplace contexts and adolescent peer relationships. Statistically significant interactions were included in our final analytical models. All analyses were conducted using STATA 16.0 (STATA Corp., College Station, TX, United States) and were adjusted for sample clustering in the survey design.

## Results

Table 1 presents descriptive statistics for the study sample. The average age of participants in 2017was 31.31 years old (std dev = 1.20). About half (52.82%) of the sample were males. The mean scores for classroom cohesion, popularity during adolescence, and academic performance were 2.99, 2.52, and 3.15, respectively.

**TABLE 1:** Descriptive characteristics of the total sample and the employed sample of young adults used in this study [percent or mean (Std Dev)], Taiwanese Youth Project.

	Percent or Mean (Std Dev)	
Variable	Total sample	The employed sample
Age (range: 30 - 33)	31.31 (1.20)	31.32 (1.14)
Male (%)	52.82	55.01
**Residence (%)**		
Taipei City	36.90	37.25
Taipei county	37.74	37.82
Yi-Lan county	25.36	24.92
*Individual characteristics in early adolescence (at aged 15)*		
**Perceived peer relation with classmates**		
Classroom cohesion (range: 1 – 4)	2.99 (0.58)	3.00 (0.57)
1. My classmates are always willing to help whenever I need them	3.02 (0.67)	3.02 (0.67)
2. I like to interact with my classmates	3.25 (0.77)	3.27 (0.76)
3. My classmates are close to each other as if we were a family	2.68 (0.80)	2.70 (0.80)
Popularity among classmates (range: 1 – 4)	2.52 (0.85)	2.53 (0.85)
Academic performance (range: 1 – 5)	3.15 (1.21)	3.16 (1.21)
Family cohesion (range: 1 – 4)	2.81 (0.65)	2.81 (0.66)
Feelings of loneliness (%)	45.89	45.47
Self-esteem (range: 1 – 4)	2.69 (0.57)	2.69 (0.57)
*Individual characteristics in adulthood (at aged 30s)*		
**Partner status (%)**		
Single, without a partner	33.57	33.58
Has a partner	26.63	27.98
Ever married	39.80	38.43
**Work characteristics**		
Currently employed (%)	90.97	
Work satisfaction (range: 1 – 4)		2.98 (0.51)
1. Working environment (range: 1 – 4)		3.01 (0.63)
2. Job duty (range: 1 – 4)		3.00 (0.56)
3. Working hours (range: 1 – 4)		2.93 (0.73)
Satisfaction of work benefits (range: 1 – 4)		2.74 (0.59)
1. Compensation/salary (range: 1 – 4)		2.79 (0.71)
2. Employee benefit (range: 1 – 4)		2.85 (0.71)
3. Promotion opportunity (range: 1 – 4)		2.57 (0.75)
Satisfying co-worker (range: 1 – 4)		3.14 (0.54)
Satisfying supervisor (range: 1 – 4)		2.97 (0.70)
Had work at night or on holiday (%)		36.25
Frequency of alcohol drinking on the job (range: 0 – 4)		0.20 (0.55)
**Loneliness clustering in adulthood (%)**		
Non-loneliness	44.13	44.08
Serious social loneliness	24.80	25.14
Severe emotional/social loneliness	31.07	30.78
N	2,520	2,287

*Std Dev = standard deviation. Percentages may not sum to 100 owing to rounding.*

About 91% of the sample reported currently being employed. Among those employed, the mean score for work satisfaction was 2.98, for benefits was 2.74, for relationships with co-workers was 3.14, and for relationships with supervisors was 2.97. About 36% had to work at night or on holidays, and the average frequency of alcohol drinking related to work was 0.20. Among this sub-sample, 44% experienced non-loneliness, while 25% experienced serious social loneliness and 31% severe emotional/social loneliness.

### Factors Associated With the Adult Loneliness Clusters

Our first multinomial logistic regression model examined the association between perceived peer relationships during adolescence and loneliness during adulthood (Table 2). The results indicated that a higher level of classroom cohesion was significantly associated with a lower relative risk of serious social loneliness or severe emotional/social loneliness, compared to non-loneliness (RRR = 0.70, 95% CI 0.58–0.85; RRR = 0.76, 95% CI 0.63–0.91, respectively). Adolescent popularity among classmates was not significantly associated with adult loneliness. Compared to non-loneliness, higher academic performance was significantly associated with a lower relative risk of serious social or severe emotional/social loneliness in adulthood (RRR = 0.87, 95% CI 0.80–0.95; RRR = 0.90, 95% CI 0.83–0.98). Feelings of loneliness during adolescence was associated with a higher relative risk of severe emotional/social loneliness in adulthood (RRR = 1.62, 95% CI 1.30–2.01). Higher adolescent self-esteem was associated with a lower relative risk of severe emotional/social loneliness in adulthood (RRR = 0.80, 95% CI 0.65–0.97).

**TABLE 2:** Multivariate multinomial logistic regression results for latent structure of loneliness among young adults, Taiwanese Youth Project (*N* = 2,520).

	Loneliness Cluster/Class Contrast	
Covariate	Serious Social Loneliness *vs.* Non-Loneliness RRR (95% CI)	Severe Emotional/Social Loneliness *vs.* Non-Loneliness RRR (95% CI)
*Individual characteristics in early adolescence (at aged 15)*		
**Perceived peer relation with classmates**		
Classroom cohesion	0.70 (0.58, 0.85)**	0.76 (0.63, 0.91)**
Popularity among classmates	1.04 (0.91, 1.19)	0.92 (0.80, 1.05)
Academic performance	0.87 (0.80, 0.95)**	0.90 (0.83, 0.98)*
Family cohesion	0.80 (0.67, 0.94)**	0.72 (0.62, 0.84)**
Feelings of loneliness	1.16 (0.92, 1.45)	1.62 (1.30, 2.01)**
Self-esteem	1.12 (0.93, 1.34)	0.80 (0.65, 0.97)*
*Individual characteristics in adulthood (at aged 30s)*		
**Partnership status (ref = Has a partner)**		
Single, without a partner	0.89 (0.69, 1.16)	1.99 (1.54, 2.56)**
Ever married	0.71 (0.56, 0.90)**	0.87 (0.68, 1.11)
Currently employed	1.04 (0.74, 1.46)	0.86 (0.62, 1.19)
Age	1.07 (0.98, 1.17)	1.11 (1.03, 1.21)**
Male	1.58 (1.28, 1.95)**	1.49 (1.21, 1.84)**
**Residence (ref = Taipei City)**		
Taipei county	1.23 (0.98, 1.55)	1.12 (0.90, 1.39)
Yi-Lan county	0.97 (0.75, 1.24)	0.82 (0.65, 1.03)

RRR = relative risk ratio; CI = confidence interval. *p < 0.05; **p < 0.01.

### Factors Associated With the Loneliness Clusters Among Employed Adults

The second part of analysis focused on the employed sample. The multivariate multinomial logistic regression model investigated the association between work-related characteristics and experiencing serious social loneliness and severe emotional/social loneliness. In addition to perceived peer relationships, we included work-related variables, while taking a wide range of individual covariates into account (Table 3). Results showed that young adults who were satisfied with their work and related benefits had lower risk of severe emotional/social loneliness. Higher levels of satisfaction with co-workers was associated with lower risk of social loneliness and severe emotional/social loneliness. The higher the frequency of alcohol drinking related to work corresponded with higher risk of serious social loneliness (RRR = 2.30, 95% CI 1.07–4.91). Interestingly, a significant interaction between frequent alcohol drinking related to work and adolescent popularity was observed (RRR = 0.67, 95% CI 0.51–0.88), indicating that young adults who reported a higher level of popularity during adolescence and frequently consumed alcohol for work had a lower risk of serious social emotional loneliness. Among the employed sub-sample, classroom cohesion and family cohesion during adolescence were still associated with loneliness during adulthood.

**TABLE 3:** Multivariate multinomial logistic regression results for work characteristics associated with latent structure of loneliness in employed young people, Taiwanese Youth Project (*N* = 2,287).

	Loneliness cluster/Class contrast	
Covariate	Serious Social Loneliness *vs.* Non-Loneliness RRR (95% CI)	Severe Emotional & Social Loneliness *vs.* Non-Loneliness RRR (95% CI)
*Individual characteristics in early adolescence (at aged 15)*		
**Perceived peer relation with classmates**		
Classroom cohesion	0.75 (0.61, 0.94)*	0.75 (0.61, 0.92)**
Popularity among classmates	1.14 (0.98, 1.34)	0.93 (0.79, 1.09)
Academic performance	0.87 (0.79, 0.96)**	0.89 (0.81, 0.98)*
Family cohesion	0.80 (0.67, 0.96)*	0.74 (0.62, 0.87)**
Feelings of loneliness	1.21 (0.96, 1.53)	1.46 (1.12, 1.89)**
Self-esteem	1.13 (0.93, 1.38)	0.83 (0.67, 1.03)
*Individual work characteristics in adulthood (at aged 30s)*		
**Work characteristics**		
Work satisfaction	0.79 (0.59, 1.05)	0.53 (0.41, 0.70)**
Satisfaction of work benefits	0.96 (0.75, 1.22)	0.79 (0.63, 0.99)*
Satisfying co-worker	0.64 (0.50, 0.81)**	0.67 (0.53, 0.85)**
Satisfying supervisor	0.94 (0.77, 1.15)	0.86 (0.71, 1.03)
Had work at night or on holiday	1.03 (0.82, 1.28)	0.89 (0.71, 1.11)
Frequent alcohol consumption for work	2.30 (1.07, 4.91)*	1.41 (0.79, 2.51)
*Interaction term*		
Popularity in early adolescence × Alcohol drinking on the job	0.67 (0.51, 0.88)**	0.89 (0.74, 1.07)
**Partnership status (ref = Has a partner)**		
Single, without a partner	0.88 (0.67, 1.16)	1.96 (1.50, 2.57)**
Ever married	0.74 (0.57, 0.94)*	0.89 (0.69, 1.16)
Age	1.06 (0.96, 1.16)	1.15 (1.05, 1.25)**
Male	1.63 (1.30, 2.04)**	1.47 (1.16, 1.86)**
**Residence (ref = Taipei City)**		
Taipei county	1.33 (1.04, 1.69)*	1.18 (0.93, 1.50)
Yi-Lan county	0.95 (0.72, 1.25)	0.90 (0.69, 1.18)

RRR = relative risk ratio; CI = confidence interval. *p < 0.05; **p < 0.01.

In addition, having ever been married was associated with a lower risk of serious social loneliness (RRR = 0.74, 95% CI 0.57–0.94). On the other hand, being single was associated with a higher risk of severe emotional and social loneliness (RRR = 1.96; 95% CI 1.50–2.57). Male gender and age were both positively associated with severe emotional and social loneliness (RRR = 1.47; 95% CI 1.16–1.86 and RRR = 1.15, 95% CI 1.05–1.25, respectively).

## Discussion

Using a large community cohort sample in Taiwan, we examined the association between perceived peer relationships in adolescence and loneliness in adulthood. We also investigated how workplace characteristics in adulthood influenced employed young adults’ risk of loneliness. Our analysis supports the literature on the existence of three clusters within the latent structure of loneliness: non-loneliness, serious social loneliness, and severe emotional/social loneliness (Hyland et al., 2018; Chiao et al., 2019; Lin and Chiao, 2020; Schwartz-Mette et al., 2020). In addition, young adults who perceived peer relationships during adolescence as positive, particularly strong classroom cohesion, had a reduced risk of social loneliness and severe social/emotional loneliness during adulthood, independent of academic performance, family background, and other psychological well-being measures, such as depressive symptomatology and self-esteem. This result partly supports the school attachment hypothesis (Dornbusch et al., 2001; Denny et al., 2011), which proposes that positive perceived peer relationships with classmates is likely to exert a protective and profound impact on psychological well-being, such that an individual is less likely to experience social and/or emotional loneliness.

Key life transitions such as graduation from school and entry into the labor market are likely to help guide researchers’ understanding of loneliness in social contexts. In workplace contexts, experiences of loneliness will vary among employed young adults. Our analysis supports prior studies (Creed and Reynolds, 2001; Wright, 2005; Gunes and Bilek, 2020), indicating that loneliness clusters are related to various dimensions of work-related characteristics, including work satisfaction, work compensation, work benefits, relationships with colleagues/supervisors, shifts worked, and alcohol consumption for work. Our findings demonstrate that both school and work contexts can influence loneliness in adulthood.

Given the development of perceived social relationships that may arise in school and in the workplace, our results indicate that satisfaction with colleagues is related to a *decreased* risk of serious social loneliness (Creed and Reynolds, 2001; Gunes and Bilek, 2020). We further examined such social relationship construction over the individual’s life course by testing the interrelationship between work-related characteristics and peer relationships in school context. Our results showed that employed adults who frequently consumed alcohol for work were more likely to experience serious social loneliness, adjusting for individual characteristics. This association may suffer from reverse causality, namely that being socially lonely leads to more alcohol drinking related to work. Notwithstanding causality, this is consistent with the hypothesis that alcohol consumption and loneliness are linked (Chiao et al., 2019; Wootton et al., 2021). However, among young adults who reported higher levels of popularity during early adolescence, drinking alcohol for work was not associated with social loneliness. Our findings suggest that alcohol drinking for work can have negative consequences for individuals’ mental health, namely, social loneliness, but can be buffered by healthy relationships in other contexts. Taiwan, similar to other East Asian countries such as Japan and South Korea, has an alcohol-drinking culture that is linked to work. This common *work tradition* remains a social norm that potentially impacts young adults’ mental health, particularly loneliness, in East Asian cultures.

Employed adults who were satisfied with their job, work compensation and benefits, were less likely to report severe emotional/social loneliness, underscoring the critical role that work satisfaction plays in experience of loneliness (Creed and Reynolds, 2001; Gunes and Bilek, 2020). Our findings also demonstrated that those who were ever married were less likely to report social loneliness compared to single adults. Specifically, single adults were more likely to report severe emotional/social loneliness, corroborating evidence for the role that social relationships play in influencing poor mental health outcomes (Chiao et al., 2019).

Interestingly, we found a gender difference in loneliness clusters. Young men were more likely than young women to report serious social loneliness during adulthood and severe emotional/social loneliness (Miething et al., 2016; Putarek and Keresteš, 2016). This suggests that men and women have a different propensity for the multiple domains of loneliness during adulthood, whereby Taiwanese men seem to be more likely than Taiwanese women to experience emotional and social loneliness during adulthood. More research is needed to explore the social, cultural, and emotional implications of gender differences in loneliness, particularly in Taiwanese society. On the other hand, status attainment theories (Blau and Duncan, 1967; Duncan, 1968) propose a causal inference on parental or early personal status and occupation in later life via macrosocial processes such as industrialization, which could be another possible social mechanism for gender differences.

There are several limitations to our study. First, attrition is important to note in this 17-year follow-up study. Attrition analysis suggested significant differences between the lost-to-follow-up (LTFU) respondents and this study’s analytical sample in terms of age, gender, and background school related factors. Firstly, subjects in the LTFU group were more likely to being older, female, and to have reported lower levels of perceived peer relationships during early adolescence. Accordingly, the association between perceived peer relationships and adult loneliness may have been underestimated. Secondly, most of the measures were self-reported, which may have been influenced by recall bias or social desirability bias. Nevertheless, the de Jong Gierveld loneliness scale did not use the term “loneliness” in its questions, the use of which may reduce the social desirability of certain answers and be associated with negative stigma. The third limitation is the use of secondary data. Information to construct loneliness clusters was only available during emerging adulthood. Future research is needed to better understand how loneliness clusters change over time and are influenced by peer relationships in adolescence. Longitudinal data on loneliness is needed to conduct a latent transition analysis model. Lastly, the sample is from Northern Taiwan and thus may not be representative of all Taiwanese youth.

## Conclusion

To our knowledge, this is a unique study on loneliness that examines a non-Western society where school and work lives occupy more than two-thirds of daily life throughout an individual’s life course. In this context, we investigated the linkage between perceived peer relationships in a school context and in the adult work environment with a latent structure of loneliness. The longitudinal data provide possible causal links between perceived peer relationships in a school context during adolescence and loneliness clusters during young adulthood. Based on a life-course perspective, our findings further suggest that the association between adult alcohol consumption for work and the risk of social loneliness depends on the individual’s level of popularity during adolescence. Our results underscore the need for understanding specific life-course factors that are associated with various domains of loneliness in order to identify appropriate interventions and policies to prevent loneliness and promote mental and social well-being (Pitman et al., 2018).

Several research questions arise from our study for future research: To what degree is poor satisfaction with workplace characteristics related to loneliness from a life course perspective? Are factors related to being in one loneliness cluster versus another similar for various family socioeconomic status groups and/or various social networks? To what degree is loneliness shaped in work contexts by levels of perceived peer relationships during adolescence? In addressing the above questions, studies will need to consider the endogeneity and reciprocal causation that may exist between work-related characteristics and the loneliness clusters.

## Data Availability Statement

The original contributions presented in this study are included in the article/supplementary material, further inquiries can be directed to the corresponding author.

## Ethics Statement

The studies involving human participants were reviewed and approved by the Research Ethics Committee of National Yang Ming Chiao Tung University (Taipei, Taiwan) (IRB Number: YM106103E-2). Written informed consent to participate in this study was provided by the participants’ legal guardian/next of kin.

## Author Contributions

CC was responsible for development of the study hypotheses, data analysis, critical revision, and finalizing of the manuscript. K-CL was responsible for development of the study hypotheses, data analysis, and drafting the manuscript. LC contributed to critical revision. All authors were involved in the writing of the manuscript, and approved the final submission.

## Conflict of Interest

The authors declare that the research was conducted in the absence of any commercial or financial relationships that could be construed as a potential conflict of interest.

## Publisher’s Note

All claims expressed in this article are solely those of the authors and do not necessarily represent those of their affiliated organizations, or those of the publisher, the editors and the reviewers. Any product that may be evaluated in this article, or claim that may be made by its manufacturer, is not guaranteed or endorsed by the publisher.

## Figures and Tables

**Appendix 1:** Latent class analysis (LCA) for conditional probabilities for the three-cluster model of loneliness among young adults using the 6-item de Jong-Gierveld short scale.

			Latent class		
	
Indicator of loneliness item			Non-loneliness	Serious social loneliness	Severe emotional and social loneliness
1. There are plenty of people I can rely on when I have problems.			0.14	0.87	0.84
2. There are many people I can trust completely.			0.10	0.94	0.87
3. There are enough people I feel close to.			0.19	0.85	0.87
4. I experience a general sense of emptiness.			0.37	0.14	0.99
5. I miss having people around.			0.77	0.65	0.94
6. I often feel rejected.			0.25	0.28	0.71
Latent class probabilities			0.44	0.25	0.31

**Model**	**L^2^**	***df***	***p*-value**	**BIC**	**AIC**

One-cluster model	2563.15	57	< 0.01	20008.44	19973.42
Two-cluster model	527.67	50	< 0.01	18027.81	17951.95
Three-cluster model	199.44	43	< 0.01	17754.44	17637.72

*AIC = Akaike’s information criterion; BIC = Bayesian information criterion.*

## Footnotes

1
http://www.typ.sinica.edu.tw
